# A New Test of Medical College Standing

**Published:** 1886-11

**Authors:** 


A NEW TEST OF MEDICAL COLLEGE STANDING.—The Illionis State Board of Health has resolved to recognize no medical college as of good standing, the aggregate of whose graduates amount to forty-five per cent. of its aggregate matriculates during a period of five years ending with any session subsequent to the session of 1885-6.

